# Removal of Azoxystrobin and Deltamethrin from Water Using Activated Biochar from *Moringa oleifera* L. Wood: Synthesis, Characterization, and Adsorption Study

**DOI:** 10.3390/molecules30132757

**Published:** 2025-06-26

**Authors:** Hiago Gomes, Ellen Bento, Maria Dayrine Tavares, Yannice Santos, José Galberto da Costa, Ronaldo do Nascimento, Stefano Salvestrini, Raimundo Teixeira

**Affiliations:** 1 Instituto Federal de Educação, Ciência e Tecnologia do Ceará, Campus Iguatu, Rodovia Iguatu/Várzea Alegre S/N, Iguatu 63500000, Brazil; hiago.gomes@ifce.edu.br; 2 Departamento de Química Biológica, Universidade Regional do Cariri, R. Cel. Antônio Luiz 1161, Crato 63105000, Brazil; ellen.bento@urca.br (E.B.); dayrine.tavares@urca.br (M.D.T.); galberto.martins@urca.br (J.G.d.C.); raimundo.teixeira@urca.br (R.T.); 3 Instituto Federal de Educação, Ciência e Tecnologia do Ceará, Campus Juazeiro do Norte, Av. Plácido Aderaldo Castelo 1646, Juazeiro do Norte 63047040, Brazil; yannice@ifce.edu.br; 4 Departamento de Físico-Química e Química Analítica, Universidade Federal do Ceará, R. Humberto Monte S/N, Fortaleza 60455700, Brazil; ronaldo@ufc.br; 5 Department of Environmental, Biological and Pharmaceutical Sciences and Technologies, University of Campania “Luigi Vanvitelli”, via Vivaldi 43, 81100 Caserta, Italy

**Keywords:** adsorption, azoxystrobin, biochar, deltamethrin, *Moringa oleifera* L., water treatment

## Abstract

The aim of this study was to evaluate the efficiency of activated biochar produced from *Moringa oleifera* L. wood for removing azoxystrobin (fungicide) and deltamethrin (insecticide) from water. The adsorption of pesticides on activated carbon was studied using batch tests evaluating the influence of contact time (1–180 min), concentration (5–50 mg L^−1^), and temperature (283, 298 and 313 K). The highest removal percentage obtained was 94.39% for azoxystrobin and 91.96% for deltamethrin, considering an initial concentration of 10 mg L^−1^ and adsorbent dosage of 5.0 g L^−1^. FTIR spectra confirmed H-bonding in the adsorption process, SEM analysis revealed homogeneous surface area characteristics, and BET results confirmed a highly superficial area for the activated carbon, all of which favor pesticide adsorption. The Boyd model showed that the intraparticle diffusion stage is determinant for both compounds in the initial stages of the adsorption process. The Sips model was the isotherm with the best fit to the experimental data, possibly indicating cooperativity between adsorbate molecules at low temperatures. The thermodynamic study showed a favorable adsorption at all the temperatures investigated, given the negative value of ΔG°. In addition, this study revealed good adsorption capacities for the material indicating that *Moringa oleifera* wood activated carbon is a viable alternative for removing azoxystrobin and deltamethrin from water.

## 1. Introduction

The massive and continuous release of pollutants in the environment poses a critical concern for the health of humans and other living organisms. Therefore, it is fundamental to develop efficient, low-cost, and sustainable techniques for removing these substances from the environment. Techniques such as biological degradation [1], advanced oxidative processes [2], and adsorption [3] can be used. However, the reuse and development of relatively low-cost green materials makes adsorption a more accessible and easily applicable method, with proven efficiency in wastewater remediation [4].

In recent years, researchers have searched for new biomaterials with high adsorption capacity to be employed in contaminated water treatment processes [5]. In this context, lignocellulosic materials have been widely used due to the high content of lignin and cellulose, which favor adsorption through functional groups such as carbonyl, carboxyl, and hydroxyl [6]. In this sense, the use of various parts of *Moringa oleifera* L. as adsorbent for contaminants of different classes is well known [6,7,8,9,10], except for wood, which has been little explored in adsorption studies. Moringa wood could exhibit good adsorption performance, since it has significant presence of hydroxyls, amines, sulfamides, and carboxylic acids [7,10,11,12]; it is also important to consider that the adsorptive capacity of the material can be increased via physicochemical modifications (in this work, pyrolysis and H_3_PO_4_ treatment), such as to produce activated carbon [13]. Activation using phosphoric acid (a non-toxic and eco-friendly solvent) results in an improvement in the characteristics and pore size of carbonaceous materials [14], even at lower temperatures than usual for pyrolysis [15], promoting reactions between the acid and the biomolecules that favor a large surface area and form new active sites (from P-O-C bonds) [16,17].

One class of pollutants worth investigating is pesticides [18]. These compounds are used extensively to improve productivity in agricultural activities, but their inappropriate and uncontrolled use leads to bioaccumulation risks and problems for human health and the environment [19,20]. Among pesticides, there are azoxystrobin (a strobilurin fungicide) and deltamethrin (a pyrethroid insecticide), which are widely used in various agricultural and livestock practices [21,22,23]. Azoxystrobin is a systemic fungicide whose activity is related to the inhibition of mitochondrial respiration in fungi [24], and is an extremely toxic substance for aquatic organisms (e.g., algae, aquatic invertebrates) [25], while deltamethrin kills insects altering their nervous system functions, and also causes serious risks to aquatic organisms such as endocrine problems, immunotoxicity, and neurotoxicity in fish [26]. In relation to human health, these compounds can cause chronic diseases in the nervous, immune, and cardiovascular systems, including carcinogenic, mutagenic, and teratogenic effects [27,28]. Recent studies show the presence of azoxystrobin in surface water [29] and deltamethrin in sediment samples [30]. In addition, in the latest report from the Program for the Analysis of Pesticide Residues in Food (PARA—Brazil), there were irregular detections (above the maximum residue limit) for both compounds in different foods [31]. Despite their adverse effects on living organisms and the presence of these compounds in the environment, little has been explored about the removal of azoxystrobin and deltamethrin from water, especially using *Moringa oleifera* derivatives as adsorbent.

In view of the above arguments, this work aims to evaluate the removal of azoxystrobin and deltamethrin from water through batch adsorption studies using activated carbon developed from *Moringa oleifera* L. wood. The adsorbent material was characterized by FTIR (Fourier Transform Infrared Spectroscopy) and SEM (Scanning Electron Microscopy) techniques. Textural properties were analyzed through adsorption/desorption of N_2_. The kinetics and equilibrium conditions were modelled and statistically validated to understand the underlying adsorption mechanism and to ensure greater reliability of the results.

## 2. Results and Discussion

### 2.1. Adsorbent Characterization

#### 2.1.1. SEM Analysis

**Figure 1:**
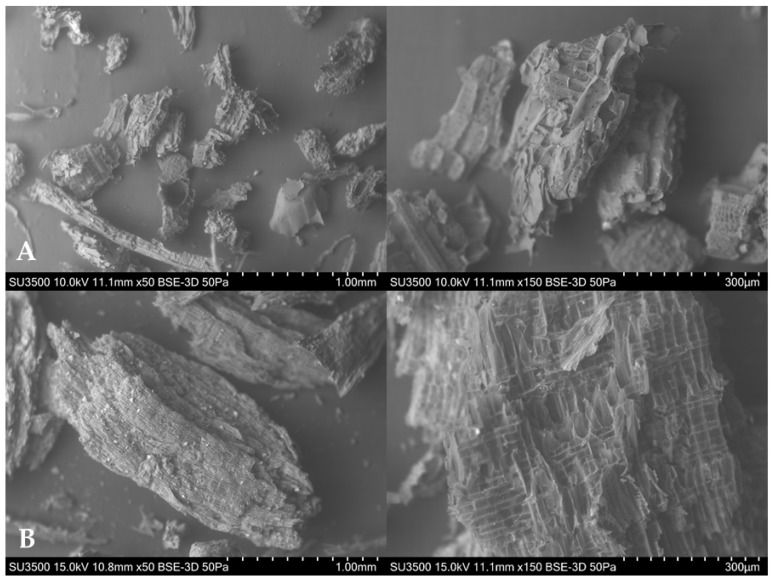
SEM analysis of *Moringa oleifera* L. wood before physicochemical modifications (**A**) and after physicochemical modifications (**B**) (1000 and 300 µm scale).

Figure 1 shows SEM images of the *Moringa oleifera* raw material (MOB) (Figure 1A) and the *Moringa oleifera* acid-activated carbon (MOB-AC) (Figure 1B).

The images clearly show that there is a significant difference between the material before and after the physicochemical modification. The morphological surface of the raw material is more heterogeneous and irregular, with a fibrous characteristic common in lignocellulosic materials. After chemical activation with phosphoric acid and physical activation by pyrolysis, the surface of the material is more homogeneous, with larger particles and a more regular structure. The changes obtained on the surface of the adsorbent material result in greater adsorption capacity for the pesticides under study, as shown later in the text.

#### 2.1.2. FTIR Analysis

**Figure 2:**
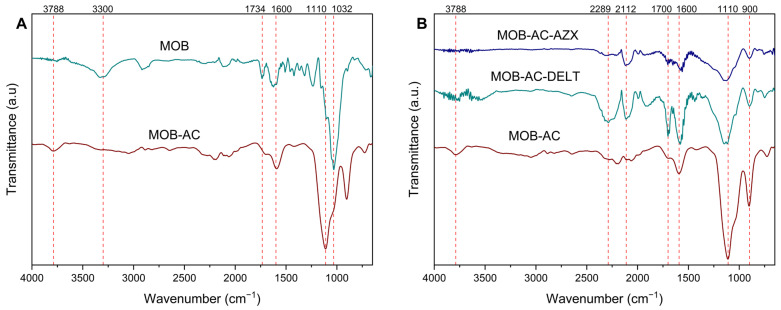
FTIR spectrum of *Moringa oleifera* L. before (MOB) and after chemical activation (MOB-AC) (**A**) and after adsorption with azoxystrobin (AZX) and deltamethrin (DELT) (**B**).

The spectra obtained by FTIR analyses are shown in Figure 2.

The FTIR spectrum of MOB shows distinctive bands that can be associated with the presence of a diversity of functional groups consistently with the characteristics of lignocellulosic materials [6]. It is also clear from Figure 2A that the chemical and physical modifications of the material (MOB-AC) alter some bands, and new bands appear. All the changes are summarized in Table S3 (Supplementary Materials).

The band at 1032 cm^−1^ observed for MOB is related to an asymmetric stretching of alkoxy groups (C-O-C), which confirms the lignin structure of Moringa [32,33]. Bands in the range between 1200 and 1000 cm^−1^ are considered fingerprint regions of lignocellulosic substances [10,34]. Also for MOB, other bands confirming the lignocellulosic structure are found at 1734 cm^−1^, which refers to the C=O stretching of carboxylic acids and esters present in hemicellulose and lignin [33,35,36], and at 1624 cm^−1^, associated with the C=C stretching vibrations of the β-(1,4)-glycosidic bonds present in cellulose [37]. Furthermore, it is possible to observe a large band at 3300 cm^−1^, which refers to hydrogen bonds of carboxylic acids, alcohols, and phenols present in lignin, cellulose, and hemicellulose [8,32,38]. This band is also related to the macromolecular association and hydrogen bonds from amines and hydroxyl groups [34].

After modification (Figure 2A, MOB-AC), the band at 3300 cm^−1^ disappears, indicating the decomposition of carboxylic acids, alcohols, and phenols due to hydrolysis caused by phosphoric acid [10]. In addition, the heat treatment brought a reduction in some bands (1734 and 1032 cm^−1^), which is possibly related to losses of lignin during the pyrolysis stage [37], highlighting a small shift in the band at 1032 to 1110 cm^−1^, after chemical and thermal treatment (Figure 2A). Among the relevant changes caused by pyrolysis and chemical activation, there is the appearance of a band at 3788 cm^−1^, which corresponds to -OH groups and chemisorbed water [10,39], a considerable increase in the peak at 900 cm^−1^, related to the beta-glycosidic bond of glucose ring of cellulose [40,41], as well as a small peak at 724 cm^−1^ corresponding to the sp^3^ carbon bonds of alkanes [5].

The changes in the chemical structures present in the material after adsorption tests with azoxystrobin and deltamethrin are shown in the FTIR results of Figure 2B. Azoxystrobin and deltamethrin molecules interacted with the functional groups present in the structure of the activated carbon. Indeed, for both the pesticides, the peak at 3788 cm^−1^ disappeared, indicating probable H bond interactions between the pesticides and the -OH bonds present in carbohydrates [4]. In addition, there was a decrease in the intensity of the peaks at 1110 cm^−1^ (asymmetric stretching of alkoxy group) and 900 cm^−1^ (β-glycosidic bond of glucose ring of cellulose). An increase in the intensity of the bands was observed at 2289 (amine group), 2115 (C=C stretch), 1919 (substituted benzene ring), 1700 (carbonyl group), and 1580 cm^−1^ (aromatic skeletal vibrations) for deltamethrin, and at 2115 cm^−1^ (C=C stretch) for azoxystrobin [42,43]. The increase in the band at 1700 cm^−1^ probably corresponds to a stretching of the carbonyl group (C=O) present in the structure of deltamethrin [42,44]. Additional evidence of the adsorbent–pesticide interaction is given by the change in the intensity ratio 1110 cm^−1^/1600 cm^−1^. The ratio changes from 0.26 for the free adsorbent to 0.68 and 1.00 for the adsorbent in the presence of azoxystrobin and deltamethrin, respectively. A significant change in the intensity of the bands is observed, indicating i) π–π interaction between alkene groups of MOB-AC and aromatic structures of the pesticides, and ii) hydrogen bonds between alkoxy groups of MOB-AC and bromine (DELT), pyrimidine (AZX), alkane groups, carbonyl, and alkoxy groups of the pesticides.

#### 2.1.3. Textural Properties Analysis

The results of the analysis of the textural properties (surface area and pore volume) of the raw material (MOB) and activated carbon (MOB-AC) are shown in Table 1.

Important changes can be seen in the structure of MOB-AC compared to MOB. The specific surface area increased significantly—about 100 times more than the raw material, as did the external surface area and the total pore volume.

At 673 K (activation temperature), phosphoric acid causes a cross-linking reaction between the acid and the biomolecule to link the hydrolyzed molecule and the phosphate into new P-O-C bonds [16], which results in P-groups added to the surface of the activated carbon that act as adsorption sites [17]. Furthermore, this temperature is not high enough to destroy the pores formed [14].

Therefore, P-activation favors pore development and an increase in surface area, where H_3_PO_4_ facilitates bond cleavage reactions from the oxidation of carbonaceous structures, forming phosphate and polyphosphate groups, and large amounts of pores [15,16], which are essential for the adsorptive capacity.

### 2.2. Kinetic Studies

**Figure 3:**
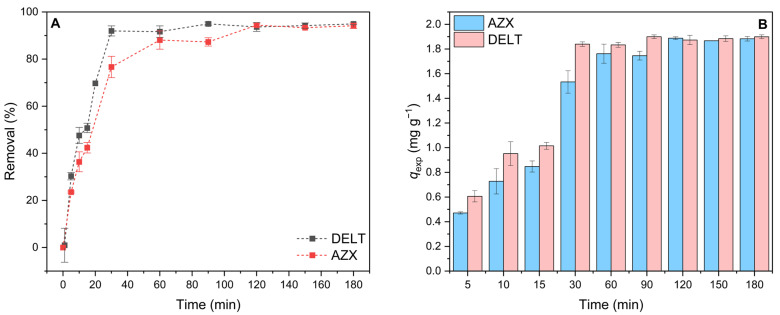
(**A**) Percentage removal and (**B**) adsorption capacity of azoxystrobin (AZX) and deltamethrin (DELT) on activated *Moringa oleifera* biochar between 0 and 180 min at 298 K, adsorbent dosage of 5.0 g L^−1^, and 10 mg L^−1^ of pesticide concentration.

The results of the kinetic experiments reveal that the activated carbon developed in this work leads to a high rate of pesticide removal in a short time (see Figure 3).

More in detail, a removal percentage of 94.39% (*q*_exp_ = 1.89 mg g^−1^, *C*_0_ = 10 mg L^−1^) was observed for azoxystrobin in 120 min, whereas for deltamethrin, a 91.96% removal efficiency (*q*_exp_ = 1.84 mg g^−1^) was achieved in 30 min. These contact times, corresponding to the highest pesticide removal (equilibrium time), were selected for the adsorption isotherm tests.

In general terms, the adsorption process of a solute on a solid surface is kinetically governed by three stages: (i) external diffusion, i.e., transport of the adsorbate through the film surrounding the adsorbent; (ii) intraparticle diffusion, i.e., transport of the adsorbate from the external surface to the pores of the adsorbent material; (iii) adsorption on the pore surface, i.e., reaction of the adsorbate on the surface of the adsorbent [45]. Thus, to evaluate the kinetic profiles in the adsorption rate, the experimental kinetic data from the adsorption tests were modelled by the PFO (pseudo-first order), PSO (pseudo-second order) and Elovich kinetic models. Table 2 shows the results of the curve fitting procedure.

From the data in Table 2, the PFO model conforms better to the experimental data for both azoxystrobin and deltamethrin, as inferred by the highest values of adjusted *R*^2^ value, as well as lowest chi-squared ($\chi^{2}$) and residual sum of squares (RSS) values. This suggests that the adsorption rate is proportional to the distance from equilibrium [46]. The graph showing the fitness of the data to the kinetic models can be found in the Supplementary Materials (Figure S1).

The better fit of the experimental data to the PFO model may be indicative of kinetics controlled by intraparticle diffusion and/or reaction at the pore surface [47]. However, the same author stated that the PFO model can be derived to assume that the kinetic mechanism can be controlled by intrafilm diffusion [48]. In addition, Plazinski [48] also stated that this model finds well-correlated data throughout the adsorption time range, while Ho and McKay [49] state that the PFO model can only be applied during the initial stages of adsorption, and the PSO model that it would be applicable for long periods.

These inconsistencies and the lack of consensus among researchers hinder efforts to determine the type of interaction in the adsorption process based on the PFO and PSO models, since multiple conclusions can be observed based on the literature. However, despite their applicability, these models continue to be the most widely used in batch adsorption studies for aqueous systems [50]. On the other hand, the Elovich model was originally proposed by Roginsky and Zeldovich in 1934 to describe the adsorption of carbon monoxide on manganese dioxide [51] and is generally used to describe processes in which chemisorption occurs on an adsorbent with a very heterogeneous surface [45].

However, as proposed by Xiao et al. [52], an erroneous conclusion can be drawn when assessing the quality of the fit of the models based solely on the *R*^2^ values. Therefore, an additional assessment of the quality of the fit of the experimental data can be made based on the residuals plot, obtained from Equation (1).

(1) $$std\;residuals=\frac{\left(q_{t}-q_{t}^{\prime }\right)}{s}$$

where $q_{t}^{\prime }$ is the amount of pesticide adsorbed at time *t* (mg g^−1^), predicted by the model (PFO, PSO, or Elovich), and *s* is the standard deviation of the residues $\left(q_{t}-q_{t}^{\prime }\right)$. If the model does indeed fit the experimental data, then the residuals should be randomly distributed around and close to zero. Otherwise, in the event of upward or downward patterns in the normalized residuals, the conclusion is that the data fit the model inadequately [53]. In Figure S2 (see Supplementary Materials), none of the models showed a random distribution of the data, which indicates a lack of fit between the kinetic models used and the experimental data obtained from the batch tests. In conclusion, the PFO kinetic model is not applicable to the data presented in the present work.

**Figure 4:**
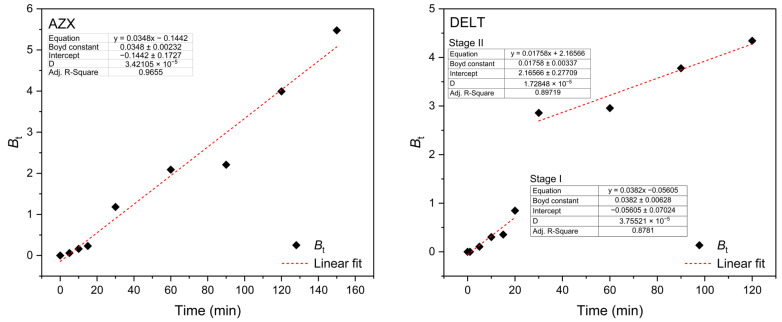
Boyd plot for the adsorption of azoxystrobin and deltamethrin on activated biochar from *Moringa oleifera* L.

The kinetic data were also fitted by using the Boyd model to assess the rate determining step in the adsorption process. In general, the reaction step on the pore surface is quite fast, which means that the diffusion step, either intrafilm or intraparticle, is the step that controls the adsorption process. In this sense, if the *B*_t_ vs. *t* graph (Figure 4) shows a linear trend passing through the origin, it can be said that the adsorption process is controlled by intraparticle diffusion [54].

The intercept value of the Boyd plot for azoxystrobin is −0.1442 ± 0.1727. The large error indicates that the parameter is indeterminable; although the plot looks linear and passes through the origin, the same is observed for deltamethrin (intercept at stage 1: −0.05605 ± 0.07024), but as the reaction time increases, the adsorption process of deltamethrin is controlled predominantly by the intrafilm diffusion (intercept at stage 2: 2.16566 ± 0.27709).

### 2.3. Adsorption Isotherms

**Figure 5:**
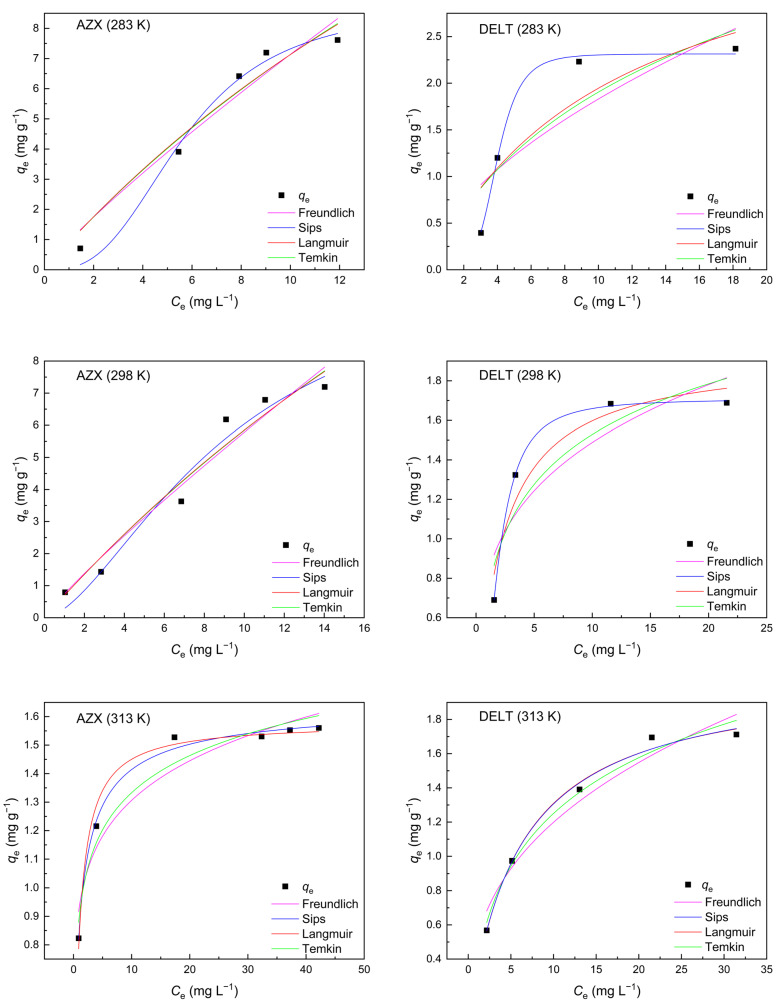
Isotherms fitting for the Freundlich, Sips, Langmuir, and Temkin models in the study of azoxystrobin (AZX) and deltamethrin (DELT) adsorption in water, using activated carbon from *Moringa oleifera* L. wood at temperatures of 283, 298, and 313 K.

Four isotherm models (Langmuir, Freundlich, Temkin, and Sips) were used to investigate the adsorption capacity and the equilibrium conditions of the activated carbons using azoxystrobin and deltamethrin. The fitted parameters calculated for each model are tabulated in Table 3. The isotherm curves can be found in Figure 5.

According to the data presented in Table 3, the Sips model showed the highest adjusted *R*^2^ values (0.947 < *R*^2^_adj_ < 0.991 for azoxystrobin; 0.992 < *R*^2^_adj_ < 0.999 for deltamethrin). The *q*_max_ values according to the Sips model were 8.756, 11.20, and 1.661 mg g^−1^ for azoxystrobin and 2.314, 1.711, and 2.931 mg g^−1^ for deltamethrin at 283, 298, and 313 K, respectively.

The Sips equation can best be used for S-shaped isotherms (sigmoidal), usually type V isotherms, which indicate a cooperative adsorption effect and can estimate the maximum adsorption capacity on a heterogeneous surface [55,56]. The exponent of the Sips model, *β*s, can then be related to the degree of cooperativity between adsorbed molecules, with the heterogeneity of the system and with the shape of the isotherm curve. When the value of *β*s is greater than the unit, heterogeneous surface and/or cooperative process might be invoked. On the other hand, when *β*s < 1, it could be evidence of negative cooperativity [57]. In Figure 5, at low temperature, the isotherms show a steeper S-shape. At 313 K, the *β*s values for azoxystrobin and deltamethrin are 0.732 and 0.654, respectively, which might be ascribed to a loss of cooperativity between the adsorbed molecules, and the isotherm is closer to the Langmuir model indicating a more uniform surface.

**Figure 6:**
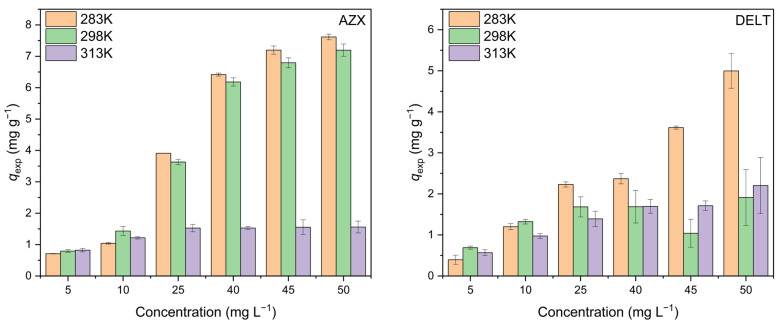
Adsorption capacity of azoxystrobin (AZX) and deltamethrin (DELT) as a function of concentration at 283, 298, and 313 K.

Figure 6 shows the adsorption capacity as a function of pesticide concentration. As the temperature rises, the adsorption capacity decreases, which might be justified by the fact that at lower temperatures there is a higher degree of cooperativity in the system.

### 2.4. Thermodynamic Studies

The adsorption thermodynamics of deltamethrin and azoxystrobin adsorption on MOB-AC was studied at 283, 298, and 313 K. The standard Gibbs free energy (Δ*G*°) values were obtained from Equation (7). The standard enthalpy (Δ*H*°) and standard entropy (Δ*S*°) values were obtained from the intercept and slope, respectively, of the straight line of the graph of ln*K*° vs. 1/*T* (Equation (8), Figure S3 (Supplementary Materials)), and the standard equilibrium constant (*K*°) was the Sips model constant, as the Sips model showed the best fit to the experimental data. The calculated thermodynamic parameters are summarized in Table 4.

The Δ*G*° values at all temperatures are negative for azoxystrobin (−17.97 to −32.58 kJ mol^−1^) and deltamethrin (−14.89 to −32.68 kJ mol^−1^), indicating that adsorption is favorable. In addition, the standard Gibbs energy becomes more negative with the increase in temperature, indicating that the adsorption process is more favorable at higher temperature [58].

Positive standard entropy values were observed for azoxystrobin (497.0 J mol^−1^ K^−1^) and deltamethrin (593.0 J mol^−1^ K^−1^). A reduction in entropy is expected during adsorption, as molecules of the adsorbate are transferred from the aqueous solution to the adsorbent. However, the entropy calculated represents the entropy of the entire process, not just adsorption. Therefore, like enthalpy, positive entropy values can be discussed in terms of secondary interactions (water–water interactions, pesticide–water interactions) [9,59].

Some authors proposing the use of *Moringa oleifera* as an adsorbent also report values of Δ*S*° > 0 and assess that positive entropy values indicate that the internal structure of the adsorbent has not been significantly altered during the adsorptive process [60], and the entropy increased due to the desolvation effect during sorbate adsorption, and the pesticides are adsorbed randomly on the surface of the adsorbent (increased randomness at the interface between adsorbent and adsorbate) [3,6,10,61,62].

The standard enthalpy values for deltamethrin and azoxystrobin adsorption were positive (123.0 kJ mol^−1^ and 152.9 kJ mol^−1^, respectively), indicating the endothermicity of the process. However, according to Escobar et al. [9] and Shahwan [63], positive Δ*H*° values show that there are secondary interactions in the adsorption process, probably due to the solvation of the adsorbate. The adsorbent–adsorbate interaction is expected to be exothermic in nature, since it refers to the energy associated with the attachment of the adsorbate molecules to the active sites of the adsorbent. However, the values reported in Table 3 are the observed standard enthalpy values, which consist of Δ*H*°_obs_ = Δ*H*°_int_ − Δ*H*°_hyd_. Δ*H*°_int_ is the intrinsic enthalpy contribution to the adsorption process, and Δ*H*°_hyd_ refers to the hydration enthalpy of the adsorbate molecule. The magnitude of Δ*H*°_hyd_ (also exothermic) can alter the sign of Δ*H*°_obs_, generating erroneous conclusions about the characteristics of the adsorptive phenomenon [3]. Thus, the fact that the standard enthalpy observed is positive does not give us the certainty of an endothermic phenomenon, but it does show that the enthalpy of hydration of the pesticide molecules is large in magnitude.

### 2.5. General Observations

Table 5 shows a comparison of the maximum adsorption capacities of different adsorbents in the removal of azoxystrobin and deltamethrin.

Both pesticides did not show the highest maximum adsorption values, but azoxystrobin showed a relatively high value compared to the work by Malhat et al. [68]. It is quite common in articles on adsorption studies to compare the maximum adsorption capacity values obtained with other studies using the same adsorbent or adsorbate. Comparisons of this nature can give rise to controversy and erroneous conclusions, since the experimental conditions (pH, adsorbent dosage, adsorbate concentration, temperature, agitation time) and the isotherm models used (linear and non-linear) need to be specified [55].

Note that the *q*_max_ value obtained in the study by Li et al. [65] is 235.4 mg g^−1^, which is quite high when compared to this study. However, that study used microplastics as adsorbent, a material with a completely different structure and functional characteristics to the lignocellulosic material in the present work. Furthermore, to avoid any bias in this discussion, it should be noted that no studies were found on azoxystrobin adsorption using lignocellulosic materials. However, even when using materials of the same nature, adsorption is a complex phenomenon whose interactions are influenced by the operational conditions used in the process.

## 3. Materials and Methods

### 3.1. Preparation of Activated Biochar from Moringa oleifera L. Wood

To prepare the activated carbon, the methodologies described by Reddy et al. [12] and Kalavathy and Miranda [10] were used with some modifications as described below. The *Moringa oleifera* wood samples were collected in the rural area of the city of Juazeiro do Norte, Ceará, Brazil. Immediately after collection, the samples were milled and then washed and dried at room temperature. The raw material was modified using chemical and thermal treatments. To do this, the sample was first mixed with a 0.1 M phosphoric acid solution in a 1:4 ratio (mass of Moringa wood/volume of phosphoric acid) and then stored for 24 h. The material was then dried in an oven at 373 K for 2 h. The chemically modified material was activated in a muffle furnace at 673 K for 1 h in an inert atmosphere (N_2_). The obtained material was named *Moringa oleifera* wood acid-activated carbon (MOB-AC).

### 3.2. Adsorbent Characterization

Scanning Electron Microscopy (SEM) was used to obtain information about the surface morphology of MOB-AC. SEM images were obtained using a SEM SU3500 (Hitachi High-Tech Corporation, Tokyo, Japan). The images were acquired in a low vacuum, with a pressure in the microscope chamber of 50 Pa, a working distance of 5.5 mm, using a backscattered electron detector (BSE-3D) (Hitachi High-Tech Corporation, Tokyo, Japan). and an electron acceleration voltage of 15 kV. Information on the chemical structures present in the adsorbent material was obtained from Fourier Transform Infrared Spectroscopy analysis using an ATR-FTIR Cary 630 (Agilent, Santa Clara, CA, USA), using an Attenuated Total Reflectance (ATR) cell equipped with a diamond crystal. Analysis of textural properties was carried out to determine the porosity and surface area of the raw material and activated carbon. A gas sorption analyzer (NOVA 800, Anton Paar, Graz, Austria) was used based on the adsorption/desorption of N_2_ at 77 K. The total surface area and pore volume were calculated using the BET (Brunauer–Emmett–Teller) equation.

### 3.3. Preparation of Pesticides Aqueous Solutions

The synthetic solutions of the pesticides were prepared from the analytical standard of azoxystrobin and deltamethrin (Sigma-Aldrich, St. Louis, MO, USA) (purity > 99.9%). Physicochemical information for both pesticides are summarized in Table S1 (Supplementary Materials). Initially, stock solutions were prepared in HPLC-grade methanol (Êxodo Científica, Sumaré, SP, Brazil) at concentrations of 2000 mg L^−1^, which were then diluted in ultrapure water to the working concentrations used in the batch adsorption experiments. All the other reagents used in the analysis were of high purity and HPLC grade and purchased from Êxodo Científica (Sumaré, SP, Brazil).

### 3.4. Batch Adsorption Studies

The batch adsorption studies were conducted using 50 mL PTFE centrifuge tubes containing known quantities of MOB-AC and pesticide solutions. The samples were agitated on a shaker table with temperature control (NT712, Nova Técnica, Piracicaba, SP, Brazil) at a speed of 100 rpm. In these studies, the effect of contact time (1–180 min), the initial concentration of the adsorbates (5–50 mg L^−1^), and the effect of temperature (283, 298, and 313 K) were evaluated.

The amount of pesticide adsorbed (*q*) at any time (*t*) was measured using Equation (2):

(2) $$q=\frac{(C_{0}-C)V}{m}$$

where *q* is the adsorption capacity (mg g^−1^); *C*_0_ and *C* are the initial concentration and the concentration at time *t*, respectively, of azoxystrobin and deltamethrin (mg L^−1^); *V* is the volume of the solution (L); and *m* is the mass of MOB-AC (g). For the batch adsorption tests, a mass of 0.05 g of the adsorbent and a solution volume of 10 mL were used.

The percentage removal of pesticides from aqueous solutions was calculated using Equation (3).

(3) $$Removal\;(\%)=\frac{\left(C_{0}-C\right)}{C_{0}}\times 100$$

#### 3.4.1. Non-Linear Kinetic and Isotherm Models

To evaluate the adsorption kinetic trend, three non-linear kinetic models were applied to the experimental data: pseudo-first order (PFO) [69], pseudo-second order (PSO) [49], and Elovich models [45]. In addition, Boyd’s intraparticle diffusion model [70] was used to evaluate the determining kinetic step in the adsorption process. The equilibrium isotherm data were modelled using the non-linear Langmuir [71], Freundlich [72], Temkin II [73,74], and Sips [75] models. Details of the kinetic and isotherm models can be found in Table S2 (Supplementary Materials).

For the Boyd kinetic model, the value of *B*_t_ (Boyd constant) was obtained from Equations (4) and (5):

(4) $$B_{t}={\pi \left(1-\sqrt{1-\frac{\pi }{3}\frac{q_{t}}{q_{e}}}\right)}^{2}$$

(5) $$B_{t}=-0.4977-ln\left(1-\frac{q_{t}}{q_{e}}\right)$$

Equation (4) should be applied for values of *q*_t_/*q*_e_ < 0.85, while Equation (5) for values of *q*_t_/*q*_e_ > 0.85 [54]. In addition, the slope obtained from the Boyd graph can be used to calculate the effective intraparticle diffusion coefficient, according to Equation (6).

(6) $$B=\frac{\pi^{2}D_{i}}{r^{2}}$$

where *B* is the slope on the Boyd graph (*B*_t_ vs. *t*), *D*_i_ is the effective intraparticle diffusion coefficient (cm^2^ min^−1^), and *r* is the radius of the adsorbent material (cm). Based on preliminary particle size tests carried out on activated carbon from *Moringa oleifera* wood, a radius of 0.0965 cm was obtained, which was considered in the calculations in Equation (6).

#### 3.4.2. Thermodynamic Parameters

The thermodynamic parameters standard adsorption Gibbs energy (Δ*G*°, kJ mol^−1^), standard adsorption entropy (Δ*S*°, J mol^−1^ K^−1^), and standard adsorption enthalpy (Δ*H*°, kJ mol^−1^) were calculated according to Equations (7) and (8) [76].

(7) ΔG°=ΔH°−TΔS°

(8) $$lnK{}^{\circ}=-\frac{\Delta H{}^{\circ}}{R}\frac{1}{T}+\frac{\Delta S{}^{\circ}}{R}$$

In Equation (8), *R* is the universal gas constant (8.3145 J K^−1^ mol^−1^) and *K*° is the thermodynamic adsorption equilibrium constant (dimensionless, obtained by expressing the solute concentration and the adsorption amount in mol L^−1^ and mol kg^−1^, respectively [76]) selected from the isotherm model that most closely matched the experimental data (e.g., *K*_L_ for the Langmuir model, *K*_T_ for the Temkin model, *K*_S_ for the Sips model).

### 3.5. Pesticide Quantification

The concentration of azoxystrobin and deltamethrin after the batch adsorption tests was obtained using high-performance liquid chromatography. A 1260 Infinity HPLC chromatograph instrument (Agilent, Santa Clara, CA, USA) coupled with UV and fluorescence detector (FLD) (Agilent, Santa Clara, CA, USA) was used. The column was an Eclipse Plus C18 (4.6 mm × 100 mm × 3.5 µm i.d.).

To detect azoxystrobin, the fluorescence detector was set at 232 nm and 315 nm for excitation and emission, respectively. As for deltamethrin, its concentration was monitored by UV detection at 210 nm.

Before the chromatographic analysis stage, the samples were prepared using the modified QuEChERS method, as described by Gomes et al. [77]. The method consists of three steps: (1) 5 mL of pesticide solution in water and 5 mL of acetonitrile were added to a 15 mL centrifuge tube and stored at −18 °C for 15 min; (2) 1.0 g of NaCl and 2.0 g of MgSO_4_ were added, followed by vortex stirring (1.0 min) and centrifugation (5.0 min, 3000 rpm); (3) 2.0 mL of the supernatant was transferred to a new centrifuge tube with 300 mg of MgSO_4_, followed by vortex stirring (1.0 min) and centrifugation (5.0 min, 3000 rpm).

## 4. Conclusions

This study evaluated the efficiency of a biochar produced from *Moringa oleifera* L. wood for removing azoxystrobin and deltamethrin from water. The morphological and functional group characteristics determined by SEM, FTIR, and BET analyses were essential to show the adsorptive potential of the biochar produced, based on a homogeneous and regular structure, with large particles, as well as polar (e.g., -OH) and apolar (e.g., -CH) functional groups. In addition, P-activation played an important role in the development of the pore structure and provided a high surface area for the activated carbon. The analysis of the residues plot showed that classical empirical kinetic models did not provide an adequate fit for the data; the Boyd model revealed that adsorption is controlled by intraparticle diffusion for both pesticides, but as the contact time increases, the predominant stage becomes intrafilm diffusion for deltamethrin. Regarding the adsorption isotherms, a better fit was obtained with the Sips model, showing that at low temperatures (283 K) the adsorbent–adsorbate cooperative interactions play a more relevant role in the adsorption process. The thermodynamic study showed that adsorption is more favorable at lower temperature. The positive Δ*H*° value indicates that the dehydration enthalpy is large enough to change the sign of Δ*H*°_obs_ (considering that intrinsic enthalpy is exothermic). The positive entropy values can be associated with the pesticides randomly adsorption on the surface of the adsorbent.

In summary, the adsorbent produced is low-cost and proves to be efficient in removing the two pesticides evaluated in this study. The few articles published on the use of *Moringa oleifera* wood as a potential adsorbent confirm the need to explore this lignocellulosic material in the removal of emerging contaminants.

## Figures and Tables

**Table 1:** Textural properties of raw *Moringa oleifera* (MOB) and activated carbon (MOB-AC).

	MOB	MOB-AC
Total surface area (m^2^ g^−1^)	1.875	188.5
External surface area (m^2^ g^−1^)	1.045	15.31
Total pore volume (cm^3^ g^−1^)	0.0036	0.0353
Average pore diameter (nm)	3.36	3.53

**Table 2:** Kinetic models for the adsorption of azoxystrobin and deltamethrin on activated carbon from *Moringa oleifera* L. wood.

Kinetic Model	Parameters	Pesticide	
		Azoxystrobin	Deltamethrin
PFO	*q*_e_ (mg g^−1^)	1.860 ± 0.036	1.901 ± 0.043
	*k*_1_	0.049 ± 0.004	0.067 ± 0.006
	Adj. *R*^2^	0.982	0.983
	$\chi^{2}$	0.00561	0.00911
	RSS	0.03928	0.09113
PSO	*q*_e_ (mg g^−1^)	2.133 ± 0.081	2.107 ± 0.087
	*k*_2_	0.027 ± 0.005	0.042 ± 0.009
	Adj. *R*^2^	0.966	0.967
	$\chi^{2}$	0.01081	0.01782
	RSS	0.07568	0.17819
Elovich	*α*	0.232 ± 0.088	0.421 ± 0.201
	*β*	2.228 ± 0.317	2.490 ± 0.387
	Adj. *R*^2^	0.917	0.922
	$\chi^{2}$	0.02658	0.04239
	RSS	0.18608	0.42393

**Table 3:** Isotherm model parameters for the adsorption of azoxystrobin and deltamethrin onto *Moringa oleifera* activated carbon.

Isotherm Model	Parameters	Azoxystrobin			Deltamethrin		
		283 K	298 K	313 K	283 K	298 K	313 K
Langmuir	*q*_max_ (mg g^−1^)	30.27	34.13	1.580	4.078	1.865	2.064
	*K*_L_ (L mg^−1^)	0.031	0.021	1.120	0.091	0.523	0.173
	Adj. *R*^2^	0.943	0.948	0.979	0.743	0.912	0.990
	$\chi^{2}$	0.47406	0.40463	0.00184	0.22239	0.01701	0.0024
	RSS	1.42217	1.6185	0.00738	0.44479	0.05103	0.0072
Freundlich	*K*_F_ (mg g^−1^ (L mg^−1^)^−1/n^)	0.953	0.744	0.934	0.481	0.906	0.512
	*n*	1.144	1.123	6.860	1.722	5.132	2.710
	Adj. *R*^2^	0.928	0.942	0.912	0.648	0.660	0.940
	$\chi^{2}$	0.59268	0.45466	0.00776	0.30372	0.06528	0.01449
	RSS	1.77803	1.81863	0.03102	0.60745	0.19585	0.04347
Temkin II	*q*_T_ (mg g^−1^)	13.89	15.93	0.188	1.417	0.294	0.500
	*K*_T_ (L mg^−1^)	0.067	0.044	119.6	0.284	13.95	1.117
	Adj. *R*^2^	0.941	0.948	0.949	0.715	0.747	0.977
	$\chi^{2}$	0.4865	0.40976	0.00448	0.24634	0.04856	0.00554
	RSS	1.45949	1.63906	0.01792	0.49269	0.14568	0.01663
Sips	*K*_S_ (L mg^−1^)	0.007	0.026	1.061	3.854 × 10^−4^	0.271	0.165
	*β*_S_	2.876	1.648	0.732	5.714	2.088	0.654
	*q*_max_ (mg g^−1^)	8.756	11.20	1.661	2.314	1.711	2.931
	Adj. *R*^2^	0.972	0.947	0.991	0.992	0.999	0.999
	$\chi^{2}$	0.2311	0.41649	7.5354 × 10^−4^	0.00705	1.3121 × 10^−4^	7.9215 × 10^−6^
	RSS	0.4622	1.24946	0.00226	0.00705	2.6243 × 10^−4^	0.0079

**Table 4:** Thermodynamic parameters for the adsorption of azoxystrobin and deltamethrin on activated carbon made from *Moringa oleifera* L. wood.

Temp (K)	*K*°	Δ*G*° (kJ mol^−1^)	Δ*H*° (kJ mol^−1^)	Δ*S*° (J mol^−1^ K^−1^)
**Azoxystrobin**				
283	2735	−17.67	123 ± 36	497 ± 122
298	10,811	−25.12		
313	428,123	−32.58		
**Deltamethrin**				
283	195	−14.89	153 ± 95	593 ± 318
298	136,462	−23.78		
313	88,361	−32.68		

**Table 5:** Comparison of *Moringa oleifera* wood activated carbon with other adsorbents for pesticide removal (considering Langmuir isotherm fit).

Pesticide	Adsorbent	*q*_max_, mg g^−1^ (Temperature)	Reference
Deltamethrin	*Moringa oleifera* wood activated carbon	4.078 (283 K)	Present study
	KOH-modified African walnut shell	57.64 (not specified)	[64]
	Oil shale ash	10.96 (298 K)	[65]
	*C. Verum* tree activated carbon	89.30 (313 K)	[66]
Azoxystrobin	*Moringa oleifera* wood activated carbon	34.13 (298 K)	Present study
	UV-aged polyethylene	235.4 (298 K)	[67]
	As-prepared silica nanoparticles	0.85 (283 K)	[68]

## Data Availability

Data available upon request.

## Supplementary Materials

The following supporting information can be downloaded at: https://www.mdpi.com/article/10.3390/molecules30132757/s1, Figure S1: Fitting the pseudo-first order (PFO), pseudo-second order (PSO) and Elovich kinetic models in the study of adsorption study of azoxystrobin (AZX) and deltamethrin (DELT) in water, using activated carbon from *Moringa oleifera* L. wood at 283 K; Figure S2: Residuals plot to check the fit of the experimental data to the pseudo-first order (PFO), pseudo-second order (PSO) and Elovich kinetic models for azoxystrobin (AZX) and deltamethrin (DELT) adsorption on activated carbon from *Moringa oleifera* L wood; Figure S3: Adjustment of the linear model of the van’t Hoff equation (lnK vs. 1/T plot) in the adsorption of azoxystrobin (AZX) and deltamethrin (DELT) on activated carbon from *Moringa oleifera* L. wood; Table S1: Physicochemical properties of azoxystrobin and deltamethrin; Table S2: Non-linear equations for kinetic and isotherm adsorption models; Table S3: Analysis of relevant peaks in the FTIR spectrum of *Moringa oleifera* (MOB) before and after physicochemical modifications (MOB-AC). References [4,5,8,10,21,22,24,25,26,27,28,29,30,31,32,34] are cited in the Supplementary Materials.
