# Supplementary material for: Removal of Azoxystrobin and Deltamethrin from Water Using Activated Biochar from Moringa oleifera L. Wood: Synthesis, Characterization, and Adsorption Study
Source: Molecules. 2025 Jun 26;30(13):2757. doi: 10.3390/molecules30132757 (PMC12251124; doi:10.3390/molecules30132757)
Supplement: Supplementary file 1 [file molecules-30-02757-s001.zip › molecules-3632830-supplementary.pdf]

molecules

MDPI

Supplementary material

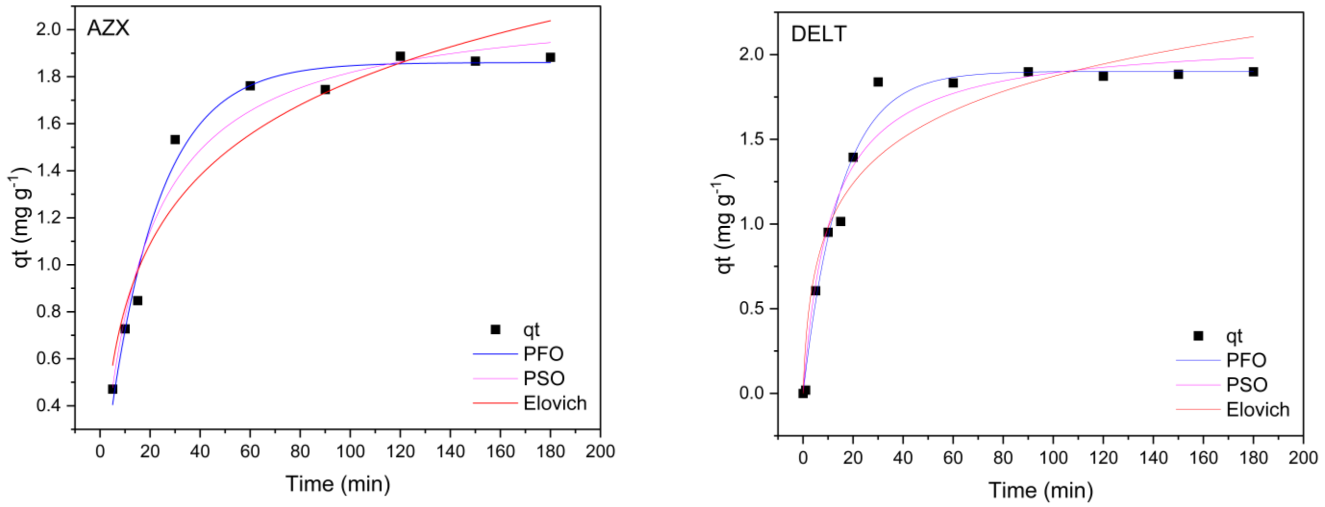

Figure S1. Fitting the pseudo-first order (PFO), pseudo-second order (PSO) and Elovich kinetic models in the study of adsorption study of azoxystrobin (AZX) and deltamethrin (DELT) in water, using activated carbon from *Moringa oleifera* L. wood at 283 K.

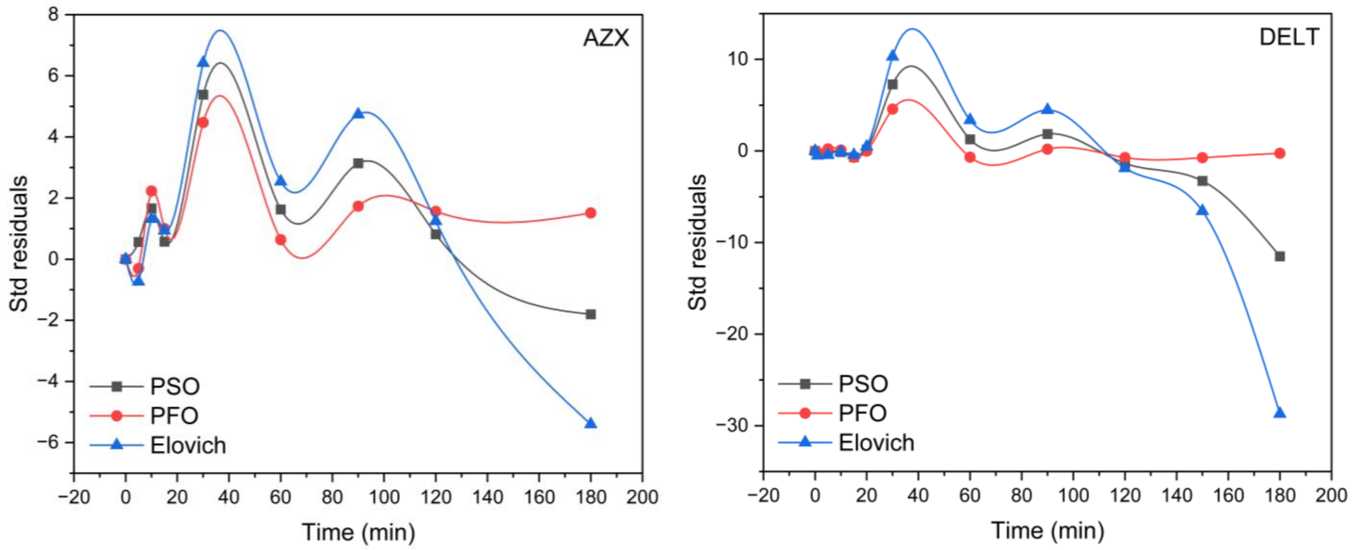

Figure S2. Residuals plot to check the fit of the experimental data to the pseudo-first order (PFO), pseudo-second order (PSO) and Elovich kinetic models for azoxystrobin (AZX) and deltamethrin (DELT) adsorption on activated carbon from *Moringa oleifera* L wood.

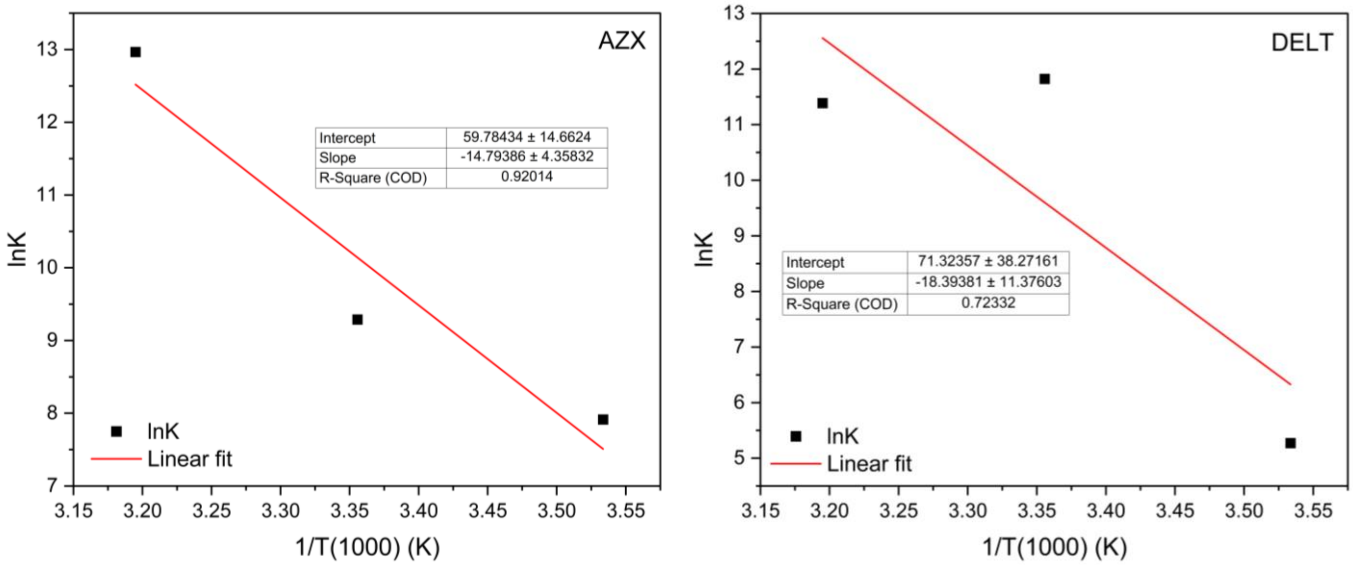

Figure S3. Adjustment of the linear model of the van't Hoff equation (lnK vs 1/T plot) in the adsorption of azoxystrobin (AZX) and deltamethrin (DELT) on activated carbon from *Moringa oleifera* L. wood.

Table S1. Physicochemical properties of azoxystrobin and deltamethrin.

	**Azoxystrobin**	**Deltamethrin**
Molecular structure	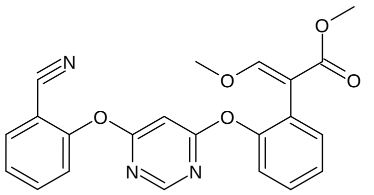	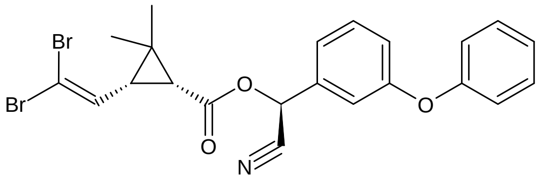
Molecular formula	$C_{22}H_{17}N_3O_5$	$C_{22}H_{19}Br_2NO_3$
IUPAC name	methyl (E)-2-{2-[6-(2-cyanophenoxy)pyrimidin-4-yloxy]phenyl}-3-methoxyacrylate	(S)-α-cyano-3-phenoxybenzyl (1R,3R)-3-(2,2-dibromovinyl)-2,2-dimethylcyclopropanecarboxylate
Chemical group (Type)	Strobilurin (Fungicide)	Pyrethroid (Insecticide)
Molar mass (g mol^{-1})	403.4	505.2
Solubility in water (mg L^{-1})	6.7 (293 K)	0.0002 (293 K)
Solubility in methanol (g L^{-1})	20 (293 K)	0.815 (293 K)

Table S2. Non-linear equations for kinetic and isotherm adsorption models.

Kinetic models	Equation	Parameters
PFO	$q_t = q_e(1 - e^{-k_1 t})$	q_t: adsorption capacity at time t (mg g^{-1})<br>q_e: adsorption capacity at equilibrium (mg g^{-1})<br>k_1: pseudo-first order adsorption rate constant (min^{-1})<br>t: time (min)
PSO	$q_t = \frac{q_e^2 k_2 t}{1 + q_e k_2 t}$	k_2: pseudo-second order adsorption rate constant (g mg^{-1} min^{-1})
Elovich	$q_t = \frac{1}{\beta}\ln(1 + (\alpha\beta t))$	α: initial adsorption rate (mg g^{-1} min^{-1}<br>β: Elovich constant (g mg^{-1})
**Isotherm models**	**Equation**	**Parameters**
Langmuir	$q_e = \frac{q_{max} K_L C_e}{1 + K_L C_e}$	q_e: adsorption capacity at equilibrium (mg g^{-1})<br>q_{max}: maximum adsorption capacity (mg g^{-1})<br>K_L: Langmuir constant (L mg^{-1})<br>C_e: adsorbate concentration at equilibrium (mg L^{-1})
Freundlich	$q_e = K_F C_e^{1/n}$	K_F: Freundlich constant (mg g^{-1} (L $mg^{-1})^{-1/n}$)<br>n: exponent of the Freundlich model (dimensionless)
Temkin II	$q_e = q_T \ln(1 + K_T C_e)$	q_T: maximum adsorption capacity according to the Temkin model (mg g^{-1})<br>K_T: Temkin constant (L mg^{-1})
Sips	$q_e = \frac{q_{max} K_S C_e^{\beta s}}{1 + K_S C_e^{\beta s}}$	K_S: Sips constant (L mg^{-1})<br>βs: exponent of the Sips model (dimensionless)

Table S3. Analysis of relevant peaks in the FTIR spectrum of *Moringa oleifera* (MOB) before and after physicochemical modifications (MOB-AC).

IR spectrum (cm^{-1})	Functional groups	References
	**MOB**	
3300	Stretching of hydroxyl groups	[8,21,27,28]
2920	Asymmetric stretching of -CH from sp^3 carbon	[21,27,28]
1734	C=O stretching of carbonyl group (ketone or aldehyde group)	[22,25,27]
1624	C=C stretching of alkene	[26,27]
1543-1365	C-O stretching of carboxylic acids, amides, and phenolic groups	[4,10]
1510	C=C aromatic vibrations of lignin	[5,34]
1460	-CH asymmetric deformation of cellulose and lignin	[30]
1421	C=C aromatic ring of lignin	[32,34]
1370	C-C stretch vibration of aromatic ring	[52]
1320	Presence of carboxylic acids	[10]
1242	Presence of carboxylic acids	[24]
1032	Asymmetric stretching (C-O-C)	[21,28]
	**MOB-AC (relevant peaks)**	
3788	-OH stretching and chemisorbed water	[8,29]
900	β-glycosidic bond of glucose ring of cellulose	[30,31]
724	sp^3 carbon bond (alkanes)	[5]
